# Light tuned to the avian eye elicits early detection and escape from an approaching aircraft

**DOI:** 10.1098/rsos.250047

**Published:** 2025-06-04

**Authors:** Ryan Bradley Lunn, Bradley Blackwell, Patrice Baumhardt, Anne Talbot, Isaac Di Domenico, Esteban Fernández-Juricic

**Affiliations:** ^1^ Biological Sciences, Purdue University, West Lafayette, IN, USA; ^2^ USDA-APHIS/WS National Wildlife Research Center, Sandusky, OH, USA

**Keywords:** bird and aircraft collisions, animal–vehicle collisions, lights, escape behaviour, flight-initiation distance, hazing

## Abstract

Collisions between birds and aircraft are a global problem. We identified different behavioural parameters affecting the probability of escape from a potential collision with an approaching aircraft, which is a function of the probability that the animal initiates an escape response (probability of reaction) and the probability of having enough time to escape (probability of sufficient time). Lights of high chromatic contrast tuned to the avian eye have been proposed as a solution to mitigate collisions. We approached Canada geese with a drone to estimate how aircraft lighting and changes in altitude, mimicking the flight phase where most strikes occur, affect parameters associated with the probability of escape. Onboard lights increased parameters associated with the probability of reaction at farther distances by promoting longer detection distances, which enabled the animal to initiate each stage of its escape response sooner leading to longer flight-initiation distances irrespective of altitude changes. Additionally, onboard lights increased parameters associated with the probability of sufficient time where longer detection distances allowed animals to escape away from (as opposed to towards) the approaching drone. Our findings have implications for the development of light technology to deter birds away from approaching vehicles, and other anthropogenic structures (such as wind turbines and solar facilities).

## 1. Introduction

Most bird populations are declining globally, and the third largest source of avian mortality is bird–vehicle collisions [1,2]. Globally, a subset of those bird–vehicle collisions occur with aircraft (hereafter, bird strikes), which also cause economic burden and safety risks for the aviation industry [3,4]. Additionally, the increase in low-altitude air operations (e.g. unoccupied aircraft systems, advanced air mobility, electrical vertical take-off and landing aircrafts) is expected to further exacerbate the bird strike problem [5,6].

One proposal towards mitigating bird strikes is the use of onboard lighting to enhance detection, potentially providing more time for the animal to initiate an escape response [7,8]. Light stimuli of high chromatic contrast to the visual system of the target species can increase detection distance due to an increase in visual conspicuousness [9] and potentially minimize the negative effects of high-speed aircraft approaches (i.e. faster approach speeds leave less time to enact a response) [10]. For onboard lighting to be effective, lights should facilitate avoidance responses regardless of aircraft flight phase. Additionally, it appears that the probability of a collision varies in different phases of flight. Specifically, most bird and aircraft collisions occur when the aircraft is descending (i.e. approach phase and landing phase) [3]. However, no experimental study of bird–aircraft collisions has examined how a change in aircraft altitude during approach affects behavioural responses, and how those responses change with and without onboard lighting [9,10].

The behavioural response of an animal to an approaching vehicle determines whether a collision occurs [11–13]. Commonly, animals rely on escape behaviour in attempting to avoid approaching vehicles [13,14]. Escape behaviour comprises a sequence or combination of behaviours beginning with detection, followed by attention allocation and threat assessment, and ending in movement away from the threat [15,16]. However, our understanding of these behaviours in the context of high-speed vehicles is limited as most studies have been focused only on the distance from the threat where the animal initiates escape (flight-initiation distance [14]).

To avoid a collision with a vehicle on a fixed trajectory, the animal must first detect and initiate a response, and that response must be quick enough to clear the trajectory of the vehicle before it arrives at the location of the animal [11,17]. We can define the *probability of escaping* as the product of the probability that the animal initiates an escape response (hereafter, *probability of reaction*), as a function of distance, and the probability of having enough time to escape given the distance at which the escape response occurred (hereafter, *probability of sufficient time*). In this study, we identified ten parameters commonly attributed to affecting the probability of escaping, and we classified them into two categories: probability of reaction (visual attention distance, detection latency, alert distance, pre-escape distance, threat display distance, flight-initiation distance and latency to flee; table 1) and probability of sufficient time (escape speed, take-off latency and probability of away trajectory; table 1).

**Table 1.** For each dependent variable considered in our analysis we provide the definition, whether it influenced the probability of reaction or the probability of sufficient time and how a change in that dependent variable might result in an increase in the probability of escape. We then included the general categorization of each behaviour as a part of the larger escape sequence and supporting citations.

variable	unit	definition	category	probability of escaping	stage	citation
visual attention distance	m	head movement behaviours associated with the allocation of attention in the direction of the approaching UAS (electronic supplementary material, table S1)	probability of reaction	positive association	detection	[18–20]
alert distance	m	distinct and overt changes in behavioural states associated with alert responses (electronic supplementary material, table S1)	probability of reaction	positive association	assessment	[21,22]
pre-escape distance	m	behaviours indicating the animal is preparing to initiate risk mitigation behaviours (electronic supplementary material, table S1)	probability of reaction	positive association	assessment and escape Initiation	[23–25]
threat display distance	m	behaviours suggesting the animal is attempting to signal and deter the UAS from approaching (electronic supplementary material, table S1)	probability of reaction	positive association/unknown	signal	[26,27]
flight-initiation distance	m	behaviours after pre-escape distance where the animal attempts to escape or mitigate risk (electronic supplementary material, table S1)	probability of reaction	positive association	escape initiation	[13,28,29]
detection latency	s	amount of time between when the UAS was first visible to when the animals first observed behavioural response occured	probability of reaction	negative association	detection	[30,31]
latency to flee	s	amount of time to initiate an escape response after becoming aware of the potential threat	probability of reaction	negative association	escape initiation	[32,33]
escape speed	m s^−1^	the average movement speed over the distance between the start and end of the escape response	probability of sufficient time	positive association	escape execution	[34,35]
take-off latency	s	amount of time between the start of the animal initiating an escape response to when first leaves the location where escape was initiated	probability of sufficient time	positive association	escape execution	[36–38]
probability of away trajectory	%	the probability of moving eastward away from the UAS (instead of towards) during the escape response	probability of sufficient time	positive association	escape execution	[39–42]

The aim of the present study was to assess whether onboard lighting technology tuned to the visual system of the viewer could improve the probability of escape (via the parameters associated with the probability of reaction and the probability of sufficient time) in wild birds at different aircraft flight phases under controlled semi-natural conditions. We approached Canada geese (*Branta canadensis*) with an unoccupied aircraft system (hereafter, UAS) varying its degree of visual conspicuousness (light-off, light-on steady, light-on pulsing), starting from different approach altitudes (level approach, descent approach) to measure the aforementioned parameters (table 1).

We selected Canada geese as our study species because they are routinely involved in damaging and costly bird strikes [43,44] due to their size and flocking behaviour. Canada goose population numbers have increased in urban areas where aircraft operations occur [43]. Therefore, understanding the escape responses of Canada geese to approaching aircraft might offer insights on how to mitigate collisions for other large bodied and social birds globally. Furthermore, the visual system of the Canada goose has been characterized [45,46], and geese tend to show avoidance responses to specific wavelengths of light [47], enabling us to test specific wavelengths that could aid geese in successfully avoiding approaching aircraft. Our findings have implications for reducing the frequency of both civil and military aircraft collisions and improving upon the application of UAS technology in a hazing context [48,49], where hazing is defined as a disturbance intended to disperse birds away from areas of anthropogenic activity [50,51].

We tested two hypotheses related to both the light and approach type treatments. First, we hypothesized that light stimuli tuned to the visual system of the viewer improves the conspicuousness of the approaching vehicle, facilitating detection at longer distances, thus providing more time to initiate each subsequent stage of the escape response [8]. We predicted that animals in response to the light-on treatments (light-on steady and light-on pulsing) relative to the light-off treatment would have longer visual attention distances, alert distances, pre-escape distances, threat display distances, flight-initiation distances, and shorter detection latency and latencies to flee (see table 1 for supporting references). Additionally, we predicted that geese would have a combination of relatively shorter take-off latencies, faster-escape speeds, and be more likely to flee away from (rather than towards; see below) the UAS (i.e. the probability of away trajectory; see below; (see table 1 for supporting references).

Second, we hypothesized that level approaches are perceived as riskier compared to descending approaches because the visual angle projected onto the retina for a descending approach is smaller upon initial detection due to a greater viewing distance [52,53]. Animals rely on the visual angle projected onto the retina to estimate distance from the approaching object and ultimately risk; where larger visual angles are associated with closer threats and thus potentially greater risk [28,52]. Consequently, we predicted that animals reacting in response to the level approach would have longer visual attention distances, alert distances, pre-escape distances, threat display distances, flight-initiation distances, but shorter detection latencies and latencies to flee relative to the descent approach. Additionally, because of the higher perceived risk associated with the level approach, we predicted that animals would have a combination of relatively shorter take-off latencies, faster escape speeds, and be more likely to flee away from (instead of towards) the UAS (i.e. probability of away trajectory; see below). We did not have an *a priori* prediction for the interaction between light and approach type.

## 2. Methods

### 2.1. Overview

We conducted our study under semi-natural conditions at the north end of Purdue Universit's Agronomy Center for Research and Education (ACRE) (40° 29′ 34.947″ N, −86° 59′ 51.1152″ W). Our study took place over the course of 55 days from 20 June to 17 August in 2023 and comprised 23 trial days. We ran trials between 06.30 and 13.00 h.

### 2.2. Animal husbandry

We collected 190 wild-caught Canada geese from Marion County, Evansville, and Scherville, IN, in collaboration with Indiana Department of Natural Resources Nuisance Waterfowl Control Operator Program [54]. Each goose was identified with a randomized combination of coloured leg bands. We housed geese between two separate facilities: a facility at the Ross Biological Reserve (6.71 m wide × 10.67 m long and 3.66 m tall) and a facility at the Animal Sciences Research and Education Center (7.62 m wide × 30.48 m long × 2.44 m tall). At both facilities, animals were provided water and food (cracked corn and Purina gamebird maintenance chow) *ad libitum*. We also provided a wide array of enrichment for the birds including pools of water, strings attached to the walls and ceilings to serve as pecking distractors, and straw bales for bedding material. In the event of serious injury or illness (i.e. 24 h or more of inactivity) animals were sedated with isoflurane and euthanized via lethal injection (1 ml per 4.5 kg of Beuthanasia) or cervical dislocation. No animals were euthanized because of this experiment. At the conclusion of this experiment individuals were retained for use in future behavioural experiments. All experimental procedures and husbandry requirements were approved by the Institutional Animal Care and Use Committee at Purdue University and overseen by Purdue Laboratory Animal Program Veterinary Staff (Purdue IACUC# 1401001019).

### 2.3. Experimental arena

**Figure 1.**
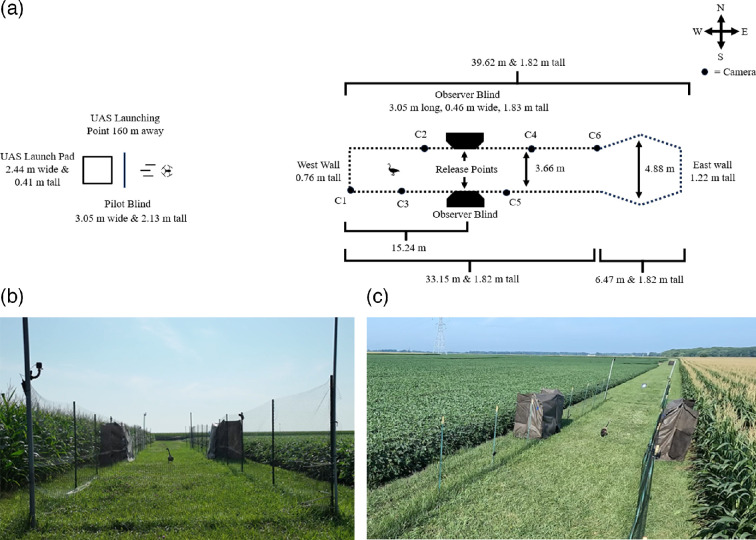
(a) Schematic design of the experimental arena. The focal animal was released into the arena from a one-way flap from the middle of either observer blind. The UAS was launched from behind a blind 160 m away. The UAS flew directly at the arena from the west and through the entirety of the arena. (b,c) Images from the UAS and camera 6 during the final seconds of trial 136 for reference.

We released a single goose per trial into a rectangular shaped experimental arena, proportionally similar to a roadway or runway (i.e. longer length and narrower width), that expanded into a hexagon shaped area at the eastern end (figure 1). The arena consisted of heavy-duty deer fencing attached to posts positioned on a grassy strip between two agricultural fields to the north and south (figure 1*a–c*). As a collision mitigation strategy, the arena had no top allowing for the UAS to easily exit by gaining altitude. The west and east sides of the arena were shorter, compared with the north and south sides, to allow for the UAS to easily enter and exit the arena at a low altitude. Attached to both the north and south sides of the arena were observation blinds, made of landscaping fabric, where the geese were initially released from. All specific measurements of the experimental arena dimensions can be found in electronic supplementary material, S1.

The UAS was launched from behind a blind 160 m away from the western end of the experimental arena by the pilot (RL, FAA Certificate Number: 4780039, Part-107, https://www.ecfr.gov/current/title-14/chapter-I/subchapter-F/part-107) (figure 1). We selected that distance because an opaque object with a width 347.5 mm, the length between the rotors of the UAS, was not theoretically resolvable to the Canada goose visual system based on visual acuity estimates [45,55,56]. Each trial was recorded from the perspective of 6, GoPro Hero 10 cameras and the UAS onboard camera all videos recorded at 60 frames per second. The location and height of the six different cameras were varied to record the entirety of the arena (electronic supplementary material, S1).

### 2.4. Behavioural experiment

Each trial day, we gathered geese from their housing enclosure, clipped their flight feathers (to prevent the animal from taking off and leaving the arena before the UAS approach started), measured their body mass, and placed each individual in a 76.2 cm long × 48.26 cm wide × 53.34 cm tall carrying crate (Top Paw Single Door Folding Wire Dog Crate) for transportation to the site of the experiment. We trimmed the flight feathers with heavy-duty 22.86 cm scissors that were sanitized with ethanol after each use. Anytime geese were being held within the carrier crates, they were given access to water *ad libitum*. While geese were waiting to receive a trial, we placed them in a shaded area (to minimize the chances of thermal stress) and 95.5 m away from the east end of the arena (to avoid visual access to the experimental arena).

We tested a single individual at a time. Before a trial began, we carried the animal in a completely covered crate to keep the animal calm and prevent the animal from seeing the arena prior to a trial. We randomized the release direction evenly between the north and south sides of the arena (94 trials released from the north, 89 trials released from the south; the different numbers were the result of some trials being excluded from the study as explained below). The goose was released into the arena through a 96.52 cm wide by 40.64 cm tall opening from behind the middle of the observer blind (figure 1). After the animal left or was prompted out of the carrier crate, cloth would fall in front of the opening so that the observers were no longer visible. We monitored the behaviour of the animal through small gaps in the blind.

Each goose was given a maximum of 15 min to settle before a trial was conducted. Once we determined that the goose was not showing aggressive behaviour (i.e. the animal was not actively pacing, hissing, running or head bobbing), and the animal was facing a westward direction, the observer signalled to the UAS pilot to launch the UAS. A trial began after the UAS was launched from behind and was no longer concealed behind the blind where the pilot was located.

We used a DJI Mavic 3 classic multi-rotor style UAS in our approaches, controlled with the DJI smart remote controller with the anti-collision lights completely covered (Dia-Jiang Innovations, Shenzhen, China). At the beginning of each trial, after ascending to the appropriate altitude and initiating forward motion, the pilot steadily increased speed until the UAS was moving at approximately 7 m s^−1^, which was determined to be the fastest speed the pilot could safely maintain through the arena. The pilot controlled the UAS from a live video feed from the DJI onboard camera that in real time reported back both UAS altitude and speed. Additionally, visual observers directly monitored the UAS during its approach through small gaps in the observation blinds.

Once the UAS was inside the arena, it continued moving forward along a straight, fixed trajectory regardless of the location of the animal until it completed the entire length of the arena; upon completion the UAS re-gained altitude to exit the eastern side of the arena (figure 1*a*). The pilot deviated the UAS only when it was necessary to avoid a collision with the goose, which was our priority, by rapidly increasing UAS altitude to exit the arena. Out of 183 trials, two UAS–goose collisions occurred. In both circumstances, the UAS came into contact with the primary feathers of the animal, both were thoroughly examined afterwards, but no injuries occurred. At the conclusion of each trial, the pilot flew the UAS back to its initial launch point, and the goose was recaptured and placed back in its carrier.

We simultaneously manipulated the light stimulus onboard the UAS (hereafter, light treatment) and the starting altitude of the UAS approach (hereafter, approach type treatment), which resulted in six unique treatment combinations. Each goose was only exposed to one of six possible combinations of light and approach type treatment.

Light treatment had three levels: light-off, light-on steady and light-on pulsing (at 2 Hz). Our light stimuli consisted of two Lume Cube RGB Panel Pro 2.0 (15.24 cm wide by 7.97 cm tall) connected with a threaded rod and attached to the UAS with a Hanatora Camera Expansion Mount Holder designed for the Mavic 3 classic. Each LED panel comprised 204 LEDs and emitted 595 lumens (approximately 931.63 W m^−2^ per our measurements; electronic supplementary material, S2)., where the lumen measurement provided is based on the manufacture’s product specifications. Both LED panels were operated with the Lume Cube control app set to display a blue colour at 100% brightness, with a peak wavelength of 457 nm (electronic supplementary material, S2).We selected this wavelength of light because blue wavelengths were found to incite a consistent avoidance response by Canada geese upon repeated exposures [47]. We also selected to test both a light steady and light pulsing treatment because previous studies had demonstrated the effects of both treatments on increasing the distance the animal first reacts [9,10].

The approach type treatment had two levels: level approach and descending approach. For a level approach, the UAS ascended above the pilot’s blind, then descended to 1 m above the ground and began approaching the arena; the pilot maintained an altitude of 1 m for the duration of the approach (i.e. a glide slope of 0°). For a descending approach, the UAS first ascended to an altitude of 8.38 m, then steadily decreased in altitude as it approached the beginning of the arena mimicking a 3° glide slope (i.e. typical landing approach of commercial aircraft) until it reached the beginning of the arena (i.e. the west side, figure 1) [57].

We measured: temperature (C), wind speed (m s^−1^), sound intensity (dB) and irradiance (μW cm^−2^ nm^−1^) as potential covariates. Temperature, wind speed and irradiance were measured at approximately goose height and at the location in the arena where the animal-initiated escape. Sound intensity was measured from camera position 3 (figure 1) per trial. Temperature and wind speed were measured with a Kestrel 3500. We did not conduct a trial if wind speed exceeded 3 m s^−1^. All values were positive regardless of wind direction.

We measured sound intensity by recording the decibel level for 2 s after the UAS entered the experimental arena, using the audio files of the video recordings of camera 3. We extracted the audio files with Adobe Audition, then measured the decibel level of each audio file with Praat speech analysis software. We estimated UAS approach speed for a given trial as the average vertical-movement-adjusted-approach speed at the instance (i.e. over a 200 ms period) of each behavioural response (visual attention, alert, pre-escape, threat, flight-initiation) (electronic supplementary material, S3). For trials 1 thorough 117, we measured absolute irradiance with an Ocean Optics, Inc. (Orlando, FL, USA) Flame-S-UV-VIS spectrometer and a P400-2-SR optical fibre with a CC-3 cosine corrector attached. However, due to equipment failure, we resorted to measuring absolute irradiance with our Ocean Insight Optics Inc. Jaz spectroradiometer, with the same optical configuration as the previous spectrometer, for trials 131 to 183.

We measured irradiance by taking two measurements with the sensor pointed in each respective cardinal direction and two additional measurements with the sensor facing directly up to the sky. To summarize the irradiance spectra per trial, we interpolated the spectral data to the nearest whole nanometer and averaged μW cm^−2^ nm^−1^ for each 1 nm interval to produce a single irradiance spectra. Each measurement ranged from 300 nm to 700 nm based on the spectrum of light visible to the avian visual system [58]. All recorded irradiance spectra can be found at OSF [59]. To analyse the effects of irradiance for each trial, we summed μW cm^−2^ for all wavelengths from 300 nm to 700 nm (the irradiance total, μW cm^−2^) as a radiometric measure of light intensity. We opted for a radiometric measure of light intensity as it is more biologically meaningful, as photometric measurements are biased by estimates of human visual perception [60].

Our study included 190 individuals gathered in the summer of 2023. Our sample size was limited primarily by the capacity of our aviaries needed to maintain high standards of animal husbandry. We did not consider seven trials due to interference that occurred during trials (i.e. animals never settling, cars driving by unexpectedly, and low-flying hawks). The final sample size for our statistical analyses was 183 individuals.

### 2.5. Video analysis

We analysed videos frame by frame with Adobe Premiere Pro. All 7 cameras (i.e. the six arena cameras and the camera onboard the UAS) were synchronized to the nearest frame using the AtomicClock: NTP Time app, whereby prior to the start of each trial time we displayed time in h:min:s:ms for each camera.

Prior to video analysis, we developed an ethogram of behaviours based on the existing literature of Canada goose behaviour, escape behaviour, and the initial observations from the pilot and observers during trials [9,13,15,61–63]. The ethogram consisted of 20 aspects related to five distinct behavioural categories: visual attention, alert, threat-display, pre-escape and flight-initiation (electronic supplementary material, S4). Importantly, the vantage of pilot from video control of the UAS and our placement of six cameras in the arena allowed us to discern between responses to the approaching UAS and potential effects of stimuli from outside the arena.

Four behavioural categories occurred sequentially (visual attention, alert, pre-escape and flight-initiation). While threat display behaviour typically occurred after alert and before flight-initiation, the animal could adopt a threat display either before or after pre-escape behaviour; therefore, we considered the behaviour separate from the sequence of the other four behaviours. Not all animals necessarily showed each behaviour in the sequence (27.33% of birds did not show all four behaviours). We measured each behaviour in the context of distance away from the UAS at the first frame the animal started showing a specific behavioural category (electronic supplementary material, S5). Again, every behaviour was corroborated with multiple camera viewpoints to ensure the behaviour of the animal was in response to the UAS.

We estimated the distance between the UAS and the goose with the UAS’s GPS tracking data and estimates of the animal’s location within the arena based on the video footage and landmarks within the arena (electronic supplementary material, S5).We measured the difference in time (s) between behaviours by multiplying the difference in frame number by the frame rate to convert the measurement to seconds (i.e. 1 s per 60 frames). Escape speed was measured as the distance between the location of the animal when it initiated escape and the location where escape ended (i.e. the animal slowed or stopped its movement) divided by the temporal difference between escape initiation and the end of escape [34,35]. In the event the animal ran into the side walls of the arena, we used that first frame the animal touched the arena netting as the termination of escape.

Detection latency was defined as the amount of time that passed between the first frame in which the UAS was visible (i.e. the start of the trial) and the first frame in which the animal displayed a behavioural response (either visual attention or alert behaviour) [30,31]. We defined latency to flee as the amount of time between the first behavioural response to the UAS and the initiation of an escape response (i.e. typically flight-initiation distance) [32,33]. Take-off latency was defined as the temporal difference between flight-initiation and the last frame the animal was touching its previous location (i.e. before leaving the ground [36,37]).

Finally, we estimated the probability of away trajectory. Specifically, we transformed escape trajectory into a binary variable (0 = the animal fled towards the UAS, 1 = the animal fled away from the UAS) [39,40,64] to overcome the challenges of applying a linear model to a circular dependent variable [65–67]. We estimated the probability using a generalized linear model (see below) to facilitate the interpretation of the results. We defined towards versus away responses based on estimates of the linear escape angle between the initial location of the animal and the location where escape ended, relative to the approaching UAS. Escape angle measurements were limited to a range between 0 and 180° [41]. Relative to the UAS being positioned at 0°, we defined a towards response as an escape angle between 0 and 90°, whereas an away response was defined as any escape angle greater than 90°. All images used to make the escape angle estimates can be found at OSF [59].

### 2.6. Statistical methods

We used R programming [68] to conduct all statistical analyses and to create our figures. Specifically, all our code was run in R version 4.3.2 except for the single general linear mixed model and data imputation which was run in R version 4.2.1 due to update incompatibilities. The code, datasets and metadata are available in electronic supplementary material, S6–S9. All other necessary files needed to reproduce this study are available at OSF [59].

We first assessed correlations between confounding variables (time of the trial, temperature, wind speed, sound intensity, irradiance, UAS speed and goose body mass) to minimize multicollinearity issues [69]. There were large correlations (i.e. *r* > 0.50) between trial time and irradiance (*r* = 0.52), trial time and temperature (*r* = 0.54), and irradiance and temperature (*r* = 0.67). Additionally, there was a large correlation between wind speed and sound intensity (*r* = 0.58).

Several studies have demonstrated that ambient light intensity affects the behavioral responses of geese light stimuli [9,47]. Due to the strong correlation between irradiance, trial time, and temperature we chose to retain irradiance and omit temperature and time of day from our analysis because of the known influence of ambient light [9,47]. Ambient sound intensity has also been shown to affect detection and escape behaviour in response to an approaching vehicle [10,70]. However, we chose to keep wind speed and remove sound intensity in our analysis for two reasons. First, our sound meter was at a fixed location within the arena, whereas the location of the animal was variable due to its movement and the size of the arena. As a result, our measure of sound intensity was not indicative of the perceived sound intensity at the location of the animal. Second, because we used the same model of UAS between trials the variation in sound between trials was likely the result of prevailing wind conditions [71]. Our intention with including wind speed as a covariate was solely to control for its potential confounding effects rather than making any type of conclusions about the effects of wind or background noise.

To test our predictions, we used general linear models to analyse the effects of light treatment and approach type treatment on our nine continuous dependent variables (table 2), and a generalized linear model to analyse the probability of away trajectory (i.e. a binary variable). Due to the sequential nature of visual attention distance, alert distance, pre-escape distance, and flight-initiation distance, we also ran a general linear mixed model to evaluate whether the distances at which animals engaged in each behavioural stage were different and to examine how much variation among stages was due to between-individual differences.

**Table 2.** General linear and generalized linear model results (significant values are bolded) for visual attention distance, alert distance, threat display distance, pre-escape distance, flight-initiation distance, escape speed, take-off latency, detection latency, latency to flee, and the probability of away trajectory. Each model for the general linear and generalized linear models included the following independent variables: light treatment, approach type treatment, goose weight, speed, irradiance, wind speed, and the interactions between light and approach type treatment, light and speed, and approach type and speed. 𝜔𝜌^2^ is a measure of effect size (partial omega squared).

general linear model results	*F*	d.f	$\omega \rho^{2}$	*p*
**visual attention distance (m) (*n* = 168**)				
*light treatment*	7.520	2155	0.074	<0.001***
*approach type treatment*	0.319	1155	−0.004	0.573
*goose weight*	0.838	1155	0.010	0.334
*speed*	26.250	1155	0.175	<0.001***
*irradiance*	0.828	1155	−0.002	0.364
*wind speed*	0.002	1155	−0.003	0.967
*light treatment × approach type treatment*	1.014	2155	0.006	0.365
*light treatment × speed*	5.417	2155	0.050	0.005**
*approach type treatment × speed*	0.142	1155	−0.005	0.707
**alert distance (m) (*n* = 183)**				
*light treatment*	1.200	2170	0.041	0.304
*approach type treatment*	1.375	1170	−0.001	0.243
*goose weight*	0.937	1170	0.012	0.334
*speed*	38.209	1170	0.461	<0.001***
*irradiance*	0.156	1170	−0.004	0.693
*wind speed*	2.224	1170	0.009	0.138
*light treatment × approach type treatment*	1.342	2170	0.004	0.264
*light treatment × speed*	0.941	2170	−0.001	0.392
*approach type treatment × speed*	2.602	1170	0.009	0.109
**threat display distance (m) log transformed (*n* = 118)**				
*light treatment*	0.051	2105	0.004	0.950
*approach type treatment*	1.871	1105	0.095	0.174
*goose weight*	0.003	1105	−0.008	0.955
*speed*	0.841	1105	−0.005	0.362
*irradiance*	0.410	1105	−0.004	0.523
*wind speed*	7.267	1105	0.061	0.008**
*light treatment × approach type treatment*	0.314	2105	−0.012	0.731
*light treatment × speed*	0.188	2105	−0.014	0.829
*approach type treatment × speed*	0.509	1105	−0.004	0.477
**pre-escape distance (m) log transformed (*n* = 148)**				
*light treatment*	0.592	2135	0.008	0.555
*approach type treatment*	0.286	1135	0.001	0.594
*goose weight*	0.572	1135	−0.005	0.451
*speed*	4.216	1135	0.119	0.042*
*irradiance*	0.185	1135	−0.003	0.668
*wind speed*	9.976	1135	0.051	0.002**
*light treatment × approach type treatment*	1.210	2135	0.003	0.302
*light treatment × speed*	0.268	2135	−0.010	0.766
*approach type treatment × speed*	0.006	1135	−0.007	0.940
**flight-initiation distance (m) log transformed (*n* = 178)**				
*light treatment*	3.692	2165	0.012	0.027*
*approach type treatment*	1.806	1165	−0.005	0.181
*goose weight*	2.858	1165	0.017	0.093
*speed*	0.136	1165	0.016	0.713
*irradiance*	1.144	1165	0.000	0.286
*wind speed*	0.045	1165	−0.005	0.832
*light treatment × approach type treatment*	3.675	2165	0.025	0.027*
*light treatment × speed*	0.048	2165	−0.011	0.953
*approach type treatment × speed*	1.905	1165	0.005	0.169
**escape speed (m s^−1^) (*n* = 168)**				
*light treatment*	1.390	2155	0.018	0.252
*approach type treatment*	0.031	1155	0.002	0.861
*goose weight*	0.011	1155	−0.006	0.916
*speed*	0.314	1155	−0.002	0.576
*irradiance*	0.431	1155	−0.003	0.512
*wind speed*	0.563	1155	−0.002	0.454
*light treatment × approach type treatment*	0.754	2155	−0.004	0.472
*light treatment × speed*	0.095	2155	−0.011	0.909
*approach type treatment × speed*	0.083	1155	−0.005	0.774
**take-off latency (ms) log transformed (*n* = 173)**				
*light treatment*	3.354	2160	−0.003	0.037*
*approach type treatment*	4.884	1160	0.016	0.029*
*goose weight*	0.972	1160	0.003	0.326
*speed*	1.225	1160	−0.002	0.270
*irradiance*	0.456	1160	−0.003	0.501
*wind speed*	0.367	1160	−0.005	0.545
*light treatment × approach type treatment*	1.776	2160	0.012	0.173
*light treatment × speed*	1.629	2160	0.007	0.199
*approach type treatment × speed*	0.126	1160	−0.005	0.723
**detection latency (s) (*n* = 183)**				
*light treatment*	7.206	2170	0.103	<0.001***
*approach type treatment*	0.002	1170	−0.003	0.960
*goose weight*	0.227	1170	0.002	0.635
*speed*	34.994	1170	0.294	<0.001***
*irradiance*	1.226	1170	0.001	0.270
*wind speed*	0.133	1170	−0.001	0.715
*light treatment × approach type treatment*	0.497	2170	−0.002	0.609
*light treatment × speed*	4.119	2170	0.032	0.017*
*approach type treatment × speed*	0.768	1170	−0.001	0.382
**latency to flee (s) (*n* = 183)**				
*light treatment*	3.914	2170	0.044	0.0218*
*approach type treatment*	3.671	1170	0.033	0.0571
*goose weight*	0.429	1170	−0.005	0.513
*speed*	27.145	1170	0.204	<0.001***
*irradiance*	0.935	1170	0.000	0.335
*wind speed*	0.078	1170	−0.003	0.781
*light treatment × approach type treatment*	0.957	2170	0.003	0.386
*light treatment × speed*	3.071	2170	0.022	0.049*
*approach type treatment × speed*	0.013	1170	−0.005	0.909

**generalized linear model**	*Χ* ^2^	d.f.	*p*	
**probability of away trajectory (*n* = 171)**				
*light treatment*	6.960	2	0.031*	
*approach type treatment*	6.277	1	0.012*	
*goose weight*	0.652	1	0.420	
*speed*	1.443	1	0.230	
*irradiance*	0.082	1	0.774	
*wind speed*	0.079	1	0.779	
*light treatment × approach type treatment*	7.950	2	0.019*	
*light treatment ×* speed	0.646	2	0.724	
*approach type treatment × speed*	1.586	1	0.208	

All general linear models and the single generalized linear model included three categorical variables (light treatment, approach type treatment, approach speed), three continuous variables (wind speed, goose weight, irradiance) and three different interaction effects (light treatment and approach type treatment, light treatment and approach speed, and approach type treatment and approach speed). We measured approach speed as a continuous variable; however, after plotting speed against each of the dependent variables, we noticed that the relationships were nonlinear, violating the linearity assumption of general linear models [72]. We decided to transform approach speed into a categorical variable to improve model fit, where speeds less than or equal to the mean observed UAS speeds (5.52 m s^−1^) were categorized as slow speeds (0.27 m s^−1^ to 5.52 m s^−1^), whereas those greater than the mean, as fast speeds (5.52 m s^−1^ to 8.09 m s^−1^). We also included body mass as a potential confounding factor because several studies have demonstrated it could affect escape behaviour [73,74]. To meet the normality of residuals and homoscedasticity assumptions of general linear models, we log-transformed threat display distance, pre-escape distance, flight-initiation distance, and take-off latency.

Unfortunately, trials 118−130 were missing irradiance data due to equipment failure in the field. To avoid information loss due to pairwise deletion, we used predictive mean matching to impute the values with the *mice* package [75]. We used temperature and time of day, given their strong correlations with irradiance, to find similar trials values but with irradiance measurements. This process generated a candidate pool of potential irradiance values for each trial with missing irradiance data. To summarize the pool of candidate values, we averaged 50 random values drawn from each candidate pool as an estimate of the average potential candidate value. We then substituted these values for the missing irradiance data. We ran our final statistical analysis with and without the imputed values to ensure they were qualitatively similar with regards to significant effects. Herein, we present the results with the imputed values (table 2) and note in our results where the results were not qualitatively similar. All model results without the imputed values are reported in electronic supplementary material, S10. The average pool of potential candidate values can be found at OSF [59].

We used the *stats* package to run both our general and generalized linear models [68]. We determined significance for each independent variable with type 3 sum of squares analysis from the *psych* package for all models [76]. Additionally, we also estimated the partial omega-squared for each independent variable as a measure of effect size. We evaluated the homogeneity of variance and normality of error assumptions for each model with the *performance* package [77]. We also used the *performance* package to implement a consensus-based approach to detect outliers [77]. We used both Cook’s distance and the minimum covariance determinant to check for outliers and chose to remove observations if both metrics deemed that an observation was an outlier; however, no outliers were identified. Whenever light treatment was significant for a given dependent variable, we utilized *t*-tests via the *emmeans* package [78] for pairwise comparisons among the three categories.

We also calculated the arithmetic means for each light treatment for all dependent variables and estimated the differences between the means of the light-on steady or light-on pulsing treatments and the mean of the light-off treatment as a measure of raw effect size (table 3). We then used a bootstrap simulation with the *Durga* package to estimate the bootstrapped confidence intervals (presented in brackets) around the differences in the means between light treatments [79]. We opted to use the arithmetic means (rather than the predicted means of the models) to inform managers of the biologically realistic effect sizes that could be used when applying our findings to potential management strategies.

**Table 3.** Sample size for each dependent variable per the three different light treatments. Effect size is the difference between the means in metres of that specific light-on treatment compared with the light off treatment and the 95% confidence intervals estimated from a bootstrap simulation of 1000 iterations.

light treatment	light-off (*n* = 63)		light-on steady (*n* = 61)		light-on pulsing (*n* = 59)
behavioural responses (*n* = 183)	*n*	*n*	effect size [95% CI]	*n*	effect size [95% CI]
*visual attention distance (m) (n = 168)*	58	56	15.07 [2.87, 30.09]	54	22.84 [10.39, 36.71]
*alert distance (m) (n = 183)*	63	61	17.26 [−1.72, 35.97]	59	19.44 [−1.20, 36.91]
*threat display distance (m) (n = 118)*	43	35	−1.078 [−5.38,10.54]	40	−1.16 [−5.1052, 2.23]
*pre-escape distance (m) (n = 148)*	49	52	14.19 [−6.27, 36.21]	47	19.17 [−6.54, 40.10]
*flight-initiation distance (m) (n = 178)*	61	58	9.74 [3.78, 20.44]	59	9.91 [3.82, 22.23]
*escape speed (m s^−1^) (n = 168)*	58	57	−0.016 [−0.59, 0.63]	53	−0.62 [−1.12, −0.03]
*take-off latency (ms) (n = 173)*	60	57	119.77 [−13.81, 361.52]	56	50.93 [−71.47, 174.67]
*probability of away trajectory (%) (n = 172)*	61	57	6.87 [−9.63, 23.61]	54	8.53 [−7.21, 24.22]
*detection latency (s) (n = 183)*	63	61	−4.69 [−7.97, −1.50]	59	−5.71 [−9.02, −2.63]
*latency to flee (s) (n = 183)*	63	61	2.10 [−1.30, 5.99]	59	5.24 [1.11, 8.85]

We ran a general linear mixed model, using the *afex* package, with behavioural category as the main independent fixed factor and distance away from the UAS for each behavioural category as the dependent variable [80]. Therefore, each individual bird was represented only once in each behavioural category. Our model considered 732 observations belonging to 183 individuals. The model also included three other fixed main factors (light treatment, approach type treatment, approach speed), and three two-way interactions (behavioural category and light treatment, behavioural category and approach type treatment, behavioural category and approach speed). The Kenward Rogers approximation was used to evaluate the significance of each independent variable for the fixed effect structure with the bound optimization quadratic approximation. The random effect structure included behavioural category as a within-subject factor and individual ID as a random factor, with random intercepts and random slopes with correlations removed to allow for model convergence. Due to the singular fit of the model we simplified the fixed structure by removing all two-way interactions [81,82] and by using the *nmkbw* optimizer.

We then estimated the marginal *R^2^* (variance attributed to just fixed effects) and conditional *R^2^* (variance attributed to both fixed and random effects) for our mixed model. We used the difference between the marginal and conditional *R^2^* estimates as a proxy of how much variation in behavioural response distance is accounted for by the random effects of the model [83,84]. We also estimated repeatability as the variance associated with between-individual differences in random intercepts and random slopes [84]. The repeatabilities of the random slopes provided an estimate of the between-individual variation in the rate of change between sequential behaviours (rather than the variation within each stage): from visual attention distance to alert distance, from alert distance to pre-escape distance and from pre-escape distance to flight-initiation distance. We then ran correlations between the repeatabilities of the intercepts and the repeatabilities of the three aforementioned slopes to determine if individuals that became aware farther away from the UAS would also tend to show positive or negative trends with changes in the different stages. Following Baker *et al*. [85], we categorized repeatability values ≤l to 20% as low individual variation, >20% or ≤40% as moderate and any score >40% as high [86].

## 3. Results

Herein, we report significant (*P* < 0.05) and non-significant (*P* ≥ 0.05) effects for each of our models. Arithmetic means and the bootstrap estimated 95% confidence intervals, presented in brackets, for both light and approach type treatments. Table 3 reports effect sizes for the light effects. Additionally, we report the results of the Tukey pairwise comparison tests for light treatment and the interactions of other factors with light treatment when significant.

### 3.1. Visual attention distance

Visual attention distance was significantly affected by light treatment (table 2), whereby the light-on pulsing treatment led to significantly longer visual attention distances (172.24 m [165.34, 177.31]) compared with the light-off treatment (149.40 m [137.16, 159.68]) (*t*_155_ = 2.57, *p* = 0.030), but without significant changes between light-on pulsing and light-on steady (164.47 m [153.71, 172.12]) (*t*_155_ = 1551.426, *p* = 0.330) and the light-on steady and light-off treatments (*t*_155_ = 1551.13, *p* = 0.495) (table 3). Approach type treatment did not significantly affect visual attention distance (level, 160.56 m [152.31, 166.98]; descending, 163.06 m [152.74, 170.97]) nor was the interaction between light and approach type treatment significant (table 2).

**Figure 2.**
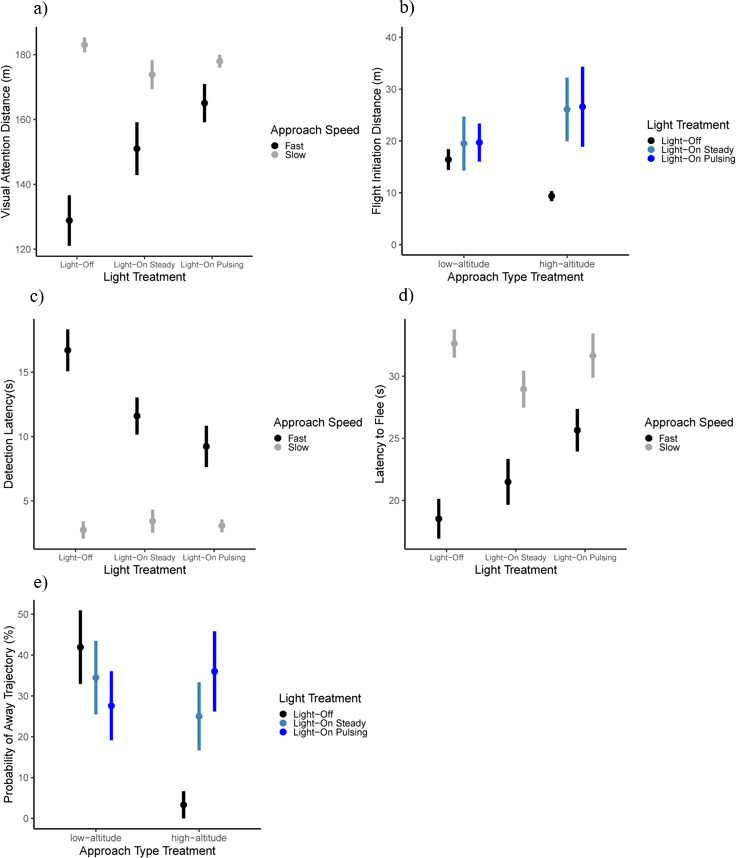
Plots of the five significant interaction effects. The circles represent the mean of the dependent variable for that specific combination of categorical variables and the error bars are their 95% confidence intervals. Plots (a), (c) and (d) show the significant interaction between light treatment and UAS approach speed for (a) visual attention distance, (c) detection latency and (d) latency to flee. Plots (b) and (e) show the significant interaction between approach type treatment and light treatment for (b) flight-initiation distance and (e) probability of away trajectory.

Approach speed significantly affected visual attention distance (table 2). When approached at slower speeds (0.27 m s^−1^ to 5.52 m s^−1^), geese initiated their visual attention response 32.23 m farther (177.69 m [171.69, 180.55]) compared with a faster approach speed (5.53 m s^−1^ to 8.09 m s^−1^) (145.46 m [135.78, 154.19]) (table 2). Further, the effect of light treatment was modulated by approach speed, as the interaction between both factors was significant (table 2; figure 2a). Specifically, visual attention distances at slow relative to fast approach speeds were significantly higher for the light-off (*t*_155_ = 6.20, *p* < 0.001) and light-on steady (*t*_155_ = 2.84, *p* = 0.005) treatments, but not for the light-on pulsing treatment (*t*_155_ = 1.66, *p* = 0.10) (figure 2a). All other effects were not significant (table 2).

### 3.2. Alert distance

Alert distance was not significantly affected by light treatment (table 2) (light-off, 123.23 m [107.18, 137.08]; light-on steady, 140.49 m [127.81, 151.21]; light-on pulsing, 142.67 m [129.48, 154.48]) (tables 2 and 3). When the imputed values for irradiance were removed, light treatment became significant (electronic supplementary material, S10). Additionally, both approach type treatments (level, 133.16 m [122.25, 142.69]; descending, 137.45 m [125.60, 148.07]) and the interaction between light and approach type treatment were not significant (table 2). Alert distance was significantly affected by approach speed (table 2). When approached at slower speeds (171.82 m [165.93, 176.05]), geese adopted an alert response to the UAS 71.96 m farther away compared with a faster approach speed (99.86 m [90.37, 109.62]). All other effects were not significant (table 2).

### 3.3. Pre-escape distance

Pre-escape distance was not significantly affected by light treatment (light-off, 73.00 m [58.30, 89.30]; light-on steady, 87.19 m [72.09, 102.55]; light-on pulsing, 92.18 m [76.42, 110.70]) (tables 2 and 3), approach type treatment (level, 79.87 m [69.52, 92.39]; descending, 88.77 m [75.13, 102.43]), nor the interaction between light and approach type treatment (table 2). Approach speed significantly affected pre-escape distances (table 2). When approached at slower speeds geese began preparing to escape 50.97 m farther from the UAS (108.87 m [94.64, 122.02]) compared with faster approach speeds (57.90 m [49.79, 67.13]). However, when the imputed values for irradiance were removed, approach speed no longer significantly affected pre-escape distance (electronic supplementary material, S10). Wind speed significantly affected pre-escape distance, whereby geese had shorter pre-escape distances with faster wind speeds (β = −0.300, SE = 0.094, based on log transformed pre-escape distance). All other effects were not significant (table 2).

### 3.4. Threat display distance

Threat display distance was not significantly affected by light treatment (light-off, 15.59 m [13.27, 19.42]; light-on steady, 14.51 m [11.02, 30.09]; light-on pulsing, 14.43 m [12.75, 17.24]) (tables 2 and 3), approach type (level, 11.26 m [10.08, 12.79]; descending, 17.54 m [15.06, 22.86]), or the interaction between light and approach type treatment (table 2). Wind speed significantly affected threat display distance, whereby geese had shorter threat display distances with faster wind speeds (β = −0.181, SE = 0.067 based on log transformed threat distance model). All other effects were not significant (table 2).

### 3.5. Flight-initiation distance

Flight-initiation distance was significantly affected by light treatment (table 2), whereby the light-on steady (22.68 m [16.87, 32.69]) and light-on pulsing (22.85 m [17.25, 33.68]) treatments led to longer flight-initiation distances than the light-off treatment (12.94 m [11.00, 15.63]) (table 3). However, the pairwise comparisons were not significantly different (light-on pulsing and light-off, *t*_165_ = 1651.50, *p* = 0.295; light-on steady and light-off, *t*_165_ = 1651.41, *p* = 0.340; light-on pulsing and light-on steady, *t*_165_ = 1650.09, *p* = 0.996). The difference in findings might be related to the *t*-statistic only considering the means between groups, as opposed to the *F*-statistic considering the ratio of the variances.

Approach type treatment did not have a significant effect on flight-initiation distance (level, 18.54 m [15.28, 24.38]; descending, 20.34 [15.49, 29.46]) (table 2). However, the interaction between light and approach type treatment was significant. For the level UAS approach there were no significant differences between light treatments (light-on pulsing versus light-off, *t*_165_ = 165−0.43, *p* = 0.90; light-on steady versus light-off, *t*_165_ = 165−0.81, *p* = 0.700; light-on pulsing versus light-on steady, *t*_165_ = 1650.38, *p* = 0.925). But for a descending approach, geese had longer flight-initiation distances with the light-on steady (*t*_165_ *=* 1652.73, *p* = 0.019) and light-on pulsing (*t*_165_ = 1652.49, *p* = 0.037) treatments compared with the light-off treatment, and without significant differences between light-on pulsing and light-on steady treatments (*t*_165_ *=* 165−0.24, *p* = 0.970) (figure 2*b*). All other effects were not significant (table 2).

### 3.6. Detection latency

Detection latency (i.e. time between UAS becoming visible to first behavioural response) was significantly affected by light treatment (table 2). Geese reacted to the UAS sooner after it first became visible for both the light-on steady (6.91 s [5.27, 9.05]) and light-on pulsing (5.89 s [4.49, 8.02]) treatments compared with the light-off treatment (11.61 s [9.10, 14.31]) (table 3). Detection latency for the light-on pulsing treatment was significantly faster than for the light-off treatment (*t*_170_ *=* −2.64, *p* = 0.025), but there were no significant differences between light-on steady and light-off (*t*_170_ *=* −1.73, *p* = 0.199) or light-on pulsing and light-on steady (*t*_170_ *=* −0.92, *p* = 0.628) treatments. Both approach types (level, 7.80 s [6.28, 9.43]; descending, 8.62 s [6.70, 10.70]) and the interaction between light and approach type did not significantly affect detection latency (table 2).

Approach speed significantly affected detection latency, where geese reacted 9.98 s sooner when approached at slower (3.13 s [2.48, 4.23]) compared to faster speeds (13.11 s [11.27, 15.15]). The interaction between light treatment and approach speed was also significant (table 2). For each light treatment the differences between slow and fast approach speeds were significant (figure 2*c*). However, those differences were greatest for the light-off treatment (light-off fast versus light-off slow, *t*_170_ = −7.32, *p* < 0.001) in that detection latency was longest for the light-off treatment when approached at a fast speed. However, the differences in latency between slow and fast approach speeds decreased in response to the light-on steady (light-on steady fast versus light-on steady slow, *t*_170_ = −4.62, *p* < 0.0001) and light-on pulsing (light-on pulsing fast versus light-on pulsing slow, *t*_170_ = −3.32, *p* = 0.001) treatments. All other effects were not significant (table 2).

### 3.7. Latency to flee

Latency to flee (i.e. time to initiate escape after the first behavioural response) was significantly affected by light treatment (table 2), whereby geese were slower to initiate escape after first reacting to the light-on pulsing treatment (28.91 s [26.35, 31.29]) compared with the light-on steady (25.77 s [23.19, 27.88]) and light-off treatments (23.67 s [20.79, 26.46]) (table 3). However, the pairwise comparisons between light treatments were not significant (light-on pulsing versus light-off, *t*_170_ *=* 1.94, *p* = 0.13; light-on steady versus light-off, *t*_170_ *=* −0.20, *p* = 0.98; light-on pulsing versus light-on steady, *t*_170_ *=* 2.14, *p* = 0.09), possibly due to the aforementioned differences with the *t-*statistic. We did not find a significant effect of approach type (level, 24.42 s [22.51, 26.23]; descending, 27.79 s [25.30, 30.06]), and the interaction between light and approach type was also not significant (table 2).

Approach speed significantly affected latency to flee (table 2); when approached at slower speeds (30.86 s [28.68, 32.53]) geese took 9.44 s longer to initiate an escape response compared with faster approach speeds (21.42 s [19.41, 23.35]). The interaction between light treatment and approach speed was also significant (table 2, figure 2*d*), where geese generally took longer to flee after detection for slow compared with fast approach speeds, but the differences between speeds were more pronounced in the light-off treatment (*t*_170_ = 6.07, *p* < 0.001) relative to the light-on steady (*t*_170_ = 3.17, *p* = 0.002) and light-on pulsing (*t*_170_ = 2.93, *p* = 0.004) treatments. All other effects were not significant (table 2).

### 3.8. Escape speed

Escape speed (i.e. movement speed after escape) was not significantly affected by light treatment (light-off, 3.12 m s^−1^ [2.79, 3.52]; light-on steady, 3.10 m s^−1^ [2.67, 3.62]; light-on pulsing, 2.50 m s^−1^ [2.13, 2.95]) (tables 2 and 3), approach type treatment (level, 3.05 m s^−1^ [2.71, 3.44]; descending, 2.77 m s^−1^ [2.48, 3.12]), nor the interaction between light treatment and approach type treatment (table 2). All other effects in the model were not significant (table 2).

### 3.9. Take-off latency

Take-off latency (i.e. time interval between the initiation and movement) was significantly affected by light treatment (table 2). Geese were slower to take-off for both the light-on pulsing (526.49 ms [448.70, 640.75]) and light-on steady treatments (595.32 ms [479.77, 819.39]) compared with the light-off treatment (475.56 ms [405.39, 565.21]) (table 3). However, all the pairwise comparisons yielded non-significant results (light-on steady and light-off, *t*_160_ *=* 0.91, *p* = 0.638; light-on pulsing and light-off, *t*_160_ *=* 0.71, *p* = 0.756; light-on pulsing and light-on steady, *t*_160_ *=* −0.19, *p* = 0.981).

Take-off latency was significantly affected by approach type, whereby geese were slower to take-off for in response to a level (568.70 ms [495.37, 675.44]) compared with a descending UAS approach (491.16 ms [416.87, 644.93]) (table 2). After removing imputed values, approach type was no longer significant (electronic supplementary material, S10).The interaction between light and approach type treatment was not significant nor were any other independent factors (table 2).

### 3.10. Probability of away trajectory

The probability of away trajectory from the UAS was significantly affected by light treatment, where geese were more likely to flee away from (instead of towards) the UAS in response to the light-on steady (29.82% [20.86.71, 38.79]) and light-on pulsing (31.48% [22.38, 40.58]) treatments compared with the light-off treatment (22.95% [14.71, 31.19]) (tables 2 and 3). However, pairwise comparisons yielded no significant differences between light treatments (light-on pulsing versus light-off, *z* = −1.87, *p* = 0.148; light-on steady versus light-off, *z* = −1.73, *p* = 0.194; light-on pulsing versus light-on steady, *z* = −0.16, *p* = 0.986). The probability of away trajectory was also significantly affected by approach type treatment, whereby geese were more likely to flee away from (instead of towards) the UAS during a level approach (34.83% [25.49, 44.17]) compared with a descending approach (20.48% [12.57, 28.39]) (table 2).

The interaction between light and approach type treatment was also significant (table 2; figure 2*e*); whereby when the UAS approached at a level altitude, there were no significant differences between light treatments (light-on pulsing versus light-off, *z* = 1.07, *p* = 0.526; light-on steady versus light-off, *z* = 0.54, *p* = 0.852; light-on pulsing versus light-on steady, *z* = 0.55, *p* = 0.848). However, for a descending UAS approach, the probability of fleeing away (instead of towards) was higher with the light-on pulsing compared with the light-off treatment (*z* = −2.61, *p* = 0.025), but no significant differences were found between the light-on steady and light-off (*z* = −2.19, *p* = 0.073) and light-on pulsing and light-on steady treatments (*z* = −0.72, *p* = 0.755). All other variables were not significant (table 2).

### 3.11. Differences in distance between behavioural stages

When considering the sequence of behavioural categories studied (visual attention distance, alert distance, pre-escape distance and flight-initiation distance), we found that distance at which animals reacted varied significantly depending on the behaviour (*F*_3,232_ = 601.99, *p* < 0.001). Specifically, visual attention distance (161.77 m [155.38, 166.56]) was longer than alert distance (135.25 m [127.42, 143.15]), alert distance was longer than pre-escape distance (84.08 m [74.86, 93.42]), and pre-escape distance which was longer than flight-initiation distance (19.40 m [16.17, 23.64]). However, light treatment (*F*_2,177_ = 2.88, *p* = 0.059; light-off, 90.02 m [80.23, 98.18]; light-on steady, 106.84 m [97.64, 117.02]; light-on pulsing, 104.09 m [95.06, 113.25]) and approach type (*F*_1,177_ = 2.50, *p* = 0.116; level, 97.84 m [90.13, 105.80]; descending, 102.72 m [95.03, 111.07]) were not significant. Only approach speed exerted a significant effect on the distance between sequential behaviours (*F*_1,177_ = 97.94, *p* < 0.001; slow, 120.74 m [112.96, 128.93]; fast, 79.69 m [73.44, 86.11]).

The fixed effects in our mixed model explained 69.8% of the variation (*R^2^* marginal), whereas the combination of both fixed and random effects explained 83.6% of the variation (*R^2^* conditional). The mixed model allowed us to explore the proportion of the variance in the random effects due to between-individual variation. Variance associated with baseline differences between individuals in visual attention distance (i.e. repeatability of the random intercepts) was 23.9%. The percentages of variance associated with between-individual differences in their transition from visual attention distance to alert distance was functionally 0%, from alert distance to pre-escape distance was 14.6%, and from pre-escape distance to flight-initiation distance was 15.1% (i.e. repeatability of the random slopes between behavioural categories).

**Figure 3.**
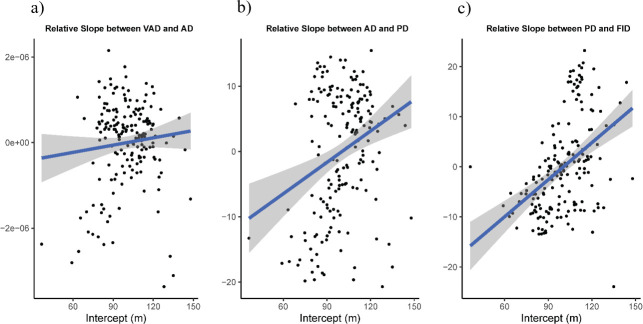
Plots of the relationship between the random intercepts and random slopes based on the random effect structure of the mixed model, considering the transitions between (a) visual attention (VAD) to alert distance (AD), (b) alert distance to pre-escape distance (PD) and (c) pre-escape distance to flight-initiation distance (FID).

We assessed if there was an association between the between-individual variation in visual attention distance and the rates of change between behavioural stages in the sequence (i.e. individuals with long visual attention distances— intercepts—have longer or shorter rates of change—slopes—between stages in the behavioural sequence). We found a low positive association between the visual attention distance intercepts and the slopes from visual attention distance to alert distance (Pearson’s correlation *r* = 0.09; figure 3*a*), and between the visual attention distance intercepts and the slopes from alert distance to pre-escape distance (Pearson’s correlation *r* = 0.28; figure 3*b*). However, we found a moderate positive association and between the visual attention distance intercepts and the slopes from pre-escape distance to flight-initiation distance (Pearson’s correlation *r* = 0.43; figure 3*c*). Overall, individuals that turned their visual attention to the UAS farther away tended to have longer alert, pre-escape and flight-initiation distances.

## 4. Discussion and conclusions

The main findings of our study suggest that a UAS fitted with a light tuned to the eyes of Canada geese (457 nm) increased the probability of reaction through increasing the distance at which birds first reacted (i.e. visual attention distance), leading the geese to initiate each stage of their escape sequence relatively sooner, ultimately resulting in an increase in flight-initiation distance. Light-on treatments also increased the probability of sufficient time by increasing the probability of away trajectory from the UAS, but the probability of sufficient time also decreased due to shorter take-off latencies (table 2, figure 2*e*). Further, the effects of light treatment on visual attention distance, detection latency and latency to flee were modulated by approach speed [10], whereas the effects of light treatment on flight-initiation distance and the probability of away trajectory were modulated by approach type (figure 2*b*,*e*). Finally, it appeared that neither light treatment nor approach type treatment had an effect on alert distance, threat display distance, pre-escape distance or escape speed.

More specifically, the UAS fitted with light-on steady and pulsing increased visual attention distance by 10.08% and 15.29%, respectively, compared with the light-off treatment (table 3). For reference, Blackwell *et al*. [9] found that on average the first alert response in a group of Canada geese (i.e. comparable with our visual attention distance) increased by 45.35% in response to a remote-controlled aircraft with a light-on pulsing treatment compared with a light-off treatment. Additionally, Blackwell *et al*. [87] found that the first alert response in a group of brown-headed cowbirds (*Molothrus ater*) approached by a truck fitted with a light increased by 3.77% and 29.24% in response to light-on pulsing (2 Hz) and the light-on steady treatments, respectively, compared with the light-off treatment. Yet, the same study [87] found the opposite response in mourning doves (*Zenaida macroura*), where the average first alert distance in a group decreased by 8.33% and 7.69% in response to a light-on pulsing and light-on steady treatment, respectively, compared with a light-off treatment. While generally, lights tuned to the eyes of the target species improves detection related behaviour [88], the trend and its magnitude are not the same for all species, which highlights the importance of how species-specific differences in physiology [70,87], sociality [89], experience [90–92], vehicle properties [93,94] and land cover [12,95,96] influence vehicle escape responses.

**Figure 4.**
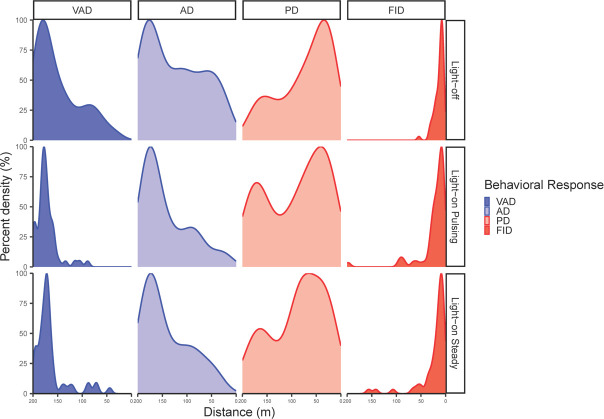
Density plots of sequential behaviours in response to different light treatments. Each plot represents the proportion of observations at different distances away from the UAS scaled to a maximum of 1. Abbreviations: VAD = visual attention distance, AD = alert distance, PD = pre-escape distance and FID = Flight-initiation distance.

The increase in visual attention distance in response to the light-on treatments allowed more time to initiate each subsequent behaviour (i.e. a cascade-effect). This increase translated into a larger relative increase in flight-initiation distance of 76.56% for the light-on pulsing treatment and 75.27% for the light-on steady treatment, compared with the light-off treatment (table 3). Further, our findings on the positive correlations between random intercepts of visual attention distance and the random slopes of the transitions across behaviours suggest that individuals with longer visual attention distances also had longer alert, pre-escape and flight-initiation distances, as has been found in other species [97]. This result suggests that an increase in detection leads to an increase in the probability of reaction at longer distances (i.e. flight-initiation distance), thus increasing the probability of escape . Figure 4 illustrates how the increase in visual attention distance cascaded throughout the escape sequence increasing the probability of reaction. For the light-on treatments, the distribution of visual attention distances was highly concentrated at farther distances with a slight left skew (reversed *x*-axis), suggesting that geese in response to the light-on treatments drew their attention to the UAS at greater distances compared with the light-off treatment where visual attention distances were more likely to occur at a variety of both longer and shorter distances (figure 4). The distribution for both alert and pre-escape distances showed a trend towards being slightly more concentrated at longer distances for the light-on treatments compared with the light-off treatment (figure 4), despite the model results being non-significant (tables 2 and 3). Lastly, the distributions of flight-initiation distances were generally right skewed for the light-on treatments, yet the distribution for the light-off treatment was exclusively concentrated at shorter distances (figure 4). The shift in distributions supports the idea that geese began each stage of their escape sequence relatively sooner in response to the light-on treatments, which could have increased the probability of reaction at a farther distance.

Light treatment affected the probability of sufficient time in two different ways. First the probability of away trajectory increased by 29.93% (table 3). Commonly, prey animals when approached directly by a threat adjust their escape trajectory to out-manoeuvre the approaching threat [39,98]. While the optimal escape trajectory might be context dependent, generally, away responses are more likely to result in the animal successfully avoiding an approaching threat [39,40,42,99,100]. Overall, the majority of geese escaped towards the approaching UAS (light-off, 22.95%; light-on steady, 29.82%; light-on pulsing, 31.48%). From the recordings, it is unclear whether animals were displaying aggressive behaviours challenging the smaller aircraft [62,101], attempting to out-manoeuvre the aircraft [39,98], or the experimental arena limited perpendicular escape angles by its narrow width (figure 1). Second, take-off latencies were 37.16% longer in response to the light-on treatments (table 3). High-speed take-offs across short distances when escaping can be metabolically costly [102]. One way animals could mitigate predation risk is to decrease their take-off latency (i.e. increase take-off velocity) [37,38], but perhaps at the cost of an increased risk of starvation over an extended period of time [103,104], suggesting animals should only adjust take-off latency when necessary. The extra time afforded by being aware of the UAS sooner likely enabled geese to execute a more informed escape response, where they increased the probability of away trajectories. However, simultaneously geese increased take-off latencies (i.e. a delay in escape) because they were likely not forced to immediately avoid a collision for the light-on treatments [32,105].

Approach type treatment also affected the probability of sufficeint time in two different ways. First, level approaches increased the probability of away trajectory from the UAS by 70.06% (i.e. increasing the probability of sufficent time) and secondly, increased take-off latency by 15.79% (decreasing the probability of sufficent time) (table 3), which might be attributed to differences in risk perception due to changes in visual angle projected onto the retina between level and descending approaches at the moment of initial detection. Animals commonly use the visual angle subtended onto the retina to determine the size and distance to an object and the rate of change in visual angle (i.e. looming) to determine when a collision might occur [52,106]. A UAS without lights approaching from the same horizontal distance but descending from a higher altitude would initially project a smaller visual angle due to the greater viewing distance relative to the visual angle of a level UAS approach. Likely, geese more readily recognized the risk associated with a larger initial visual angle approaching and began to escape sooner resulting in an adjusted escape direction away from the UAS but also simultaneously increased take-off latency [35]. This finding emphasizes that geese relied on changes in escape trajectory (i.e. probability of away trajectory) and only adjusted take-off latency when necessary as the UAS approached closer to increase the probability of sufficient time.

For the light-off treatment, geese had longer visual attention distances and briefer detection latencies when the UAS was moving at a slow compared with a fast approach speed. This decrease in detection latency is likely the result of slow speeds providing more time for the animal to process the threat at greater distances relative to a faster approach speed (figure 2a) [15,16]. However, for the light-on treatments, particularly the light-on pulsing treatment, reduced differences for both visual attention distance and detection latency between slow and fast approach speeds (figure 2*a*). This finding suggests that the light-on treatments mitigated the negative consequences of approach speed, likely due to an increase in visual conspicuousness that increased the distance at which the UAS could first be detected, thus allowing more time overall for the UAS to capture the visual attention of the animal [107,108]. Doppler *et al.* [10] found a similar trend in brown-headed cowbirds, whereby their alert responses to an approaching RC aircraft were attenuated by a light-on pulsing treatment and eliminated by a light-on steady treatment. Our results suggest that for both the light-on steady and light-on pulsing treatments geese were aware of the approaching UAS sooner even if it had already began approaching at a faster speed, which subsequently enabled geese to increase the probability of reaction at longer distances.

Latency to flee (i.e. time elapsed from first observed behavioural response to when the animal-initiated escape) was modulated by the interaction between light treatment and approach speed. For the light-off treatment, geese had briefer latencies to flee when the UAS was approaching at a fast compared with a slow speed (figure 2*d*). Faster approach speeds are associated with greater perceived risk, less time to process and respond, and thus briefer latencies to flee [109]. However, during the light-on treatments, primarily the light-on pulsing treatment, escape latencies were longer and the differences in latency to flee between fast and slow approach speeds were smaller, albeit still significantly different between speeds (figure 2d). Interestingly though, geese in response to the light-on treatments showed longer visual attention distances and longer flight-initiation distances, despite longer latencies to flee (table 3). We would expect latency to flee to vary if either variable alone changed (i.e. an increase in visual attention distance could result in an increase in latency to flee, or an increase in flight-initiation distance could result in a decrease in latency to flee). However, what we found is that both visual attention distance and flight-initiation distance increased simultaneously, but at different magnitudes, resulting in a net increase in latency to flee. Specifically, the light-on treatments led to a larger increase in visual attention distance (18.96 m) compared to the increase in flight-initiation distance (9.83 m) (table 3). This finding suggests that geese becoming aware of the threat sooner and consequently lengthen the latency to flee to further assess risk about the approaching threat, resulting in a delayed escape [17,32]. In essence, earlier visual detection allows for longer periods to process the threat before initiating escape but also resulted in longer flight-initiation distances. Generally, a longer latency to flee will reduce the probability of escaping because the more time that elapses prior to the animal initiating escape (i.e. a decrease in the probability of reaction) results in the threat getting closer, consequently decreasing the probability of sufficient time. However, latency to flee must be understood within the context of when the animal first becomes aware of the approaching threat. If the animal detects the threat at a longer distance, then a longer latency to flee might have an inconsequential effect on the probability of sufficient time.

The effects of light treatment on flight-initiation distance and the probability of away trajectory from the UAS were modulated by approach type. During level approaches, the differences between light-off and both light-on treatments were minimal (figure 2*b*,*e*). But during descending approaches, geese increased both flight-initiation distance and the probability of away trajectory from the UAS in response to the light-on treatments compared with the light-off treatment (figure 2*b*,*e*). During a descending approach with the light-off treatment, the UAS generated a smaller visual angle which might have limited detection, with out the aid of the light. However, for descending approaches coupled with the light-on treatments, detection of the approaching object was no longer limited to just the angular size of the UAS, as the light provided additional visual cues, such as a light intensity and chromatic contrast [10,110]. As such, lighting facilitated greater awareness of the UAS, prompting the animal to initiate its escape sequence sooner resulting in an increase in the probability of escaping through both increasing the probability of reaction (i.e. flight-initiation distance) and probability of sufficient time (i.e. probability of away trajectory).

Commonly, differences in escape behaviour are attributed to between-individual variation [111,112]. For a stimulus to be an effective tool to mitigate collisions it should consistently elicit similar escape behaviours regardless of the individual [113,114]. We found that between-individual differences (i.e. repeatability of the random intercepts) accounted for a low to moderate (23.9%) level of variation in visual attention behaviour. These levels of between-individual differences appear to be typical for birds (mean ± SD repeatabilities, 22.5 ± 13.4%: *Molothrus ater*, 27% [91]; *Aptenodytes patagonicus,* 10% [115]; *Anser anser*, 45% [116]; *Tringa totanus*, 21% [117]; *Aphelocoma coerulescens*, 24% [118]; *Petrochelidon pyrrhonota*, 8%, [119]). The implication is that we would expect light stimuli onboard an approaching aircraft to elicit relatively consistent changes in goose behaviour regardless of the individual.

Our results have four implications for the use of lighting technology as means of reducing bird–aircraft collisions, collisions with anthropogenic structures, and also in potential hazing applications. First, an increase in detection as the result of onboard lighting can offset the negative consequence of approach speed. Aircraft speed is a major contributing factor in the context of bird–aircraft collisions [3,120]. Bird escape responses appear inadequate when approached at extremely fast approach speeds because typically the animal has little time remaining to clear the vehicles trajectory after threat detection occurs [11,17]. Our study is the second (see [10]) to find that onboard lights can mitigate or offset the negative consequences of fast aircraft approach speeds.

Second, onboard lighting resulted in longer flight-initiation distances and higher probability of an away trajectory when the aircraft was descending. Our results are similar to what others have found: descending aircraft without lights on are less likely to prompt the initiation of escape [121,122]. However, with lights onboard, goose escape behaviour was similar for both level and descending approaches. Lights might be particularly effective at helping birds initiate the proper response to aircraft changing altitude during different flight phases, which might be particularly beneficial for rotorcraft that change altitude drastically.

Third, the intensity of our light stimuli was equivalent to a 75-watt light bulb. That intensity was sufficient to increase Canada goose detection and escape responses to a small approaching UAS. For perspective, the typical landing light onboard an approaching aircraft potentially produces 634 times more light than the LED panel used in our study [123]. Consequently, the differences in goose behavior between light treatments was caused by a relatively dim light. Potentially integrating wavelengths of high visual contrast with the existing intensity of aviation lights could perhaps further increase the detection and escape responses, but additional testing is needed.

Fourth, UAS and onboard lighting systems paired together could increase the range at which UAS operations disturb or influence the behaviour of a target species. UAS wildlife hazing often takes place at lower altitudes, which can be dangerous for both wildlife (i.e. a higher risk of collision with the UAS) and equipment (i.e. more obstacles to avoid). Based on our results, we suggest that fitting a UAS with lights tuned to the avian eye can enhance its ability to elicit escape responses when approaching from a relatively higher altitude and descending upon the animal, in turn reducing the chances of causing harm to the animals and equipment. Additionally, this technology could be applied to deter bird from wind turbines, buildings and powerlines, which are other structures with which birds collide with [124–126].

## Data Availability

All data, files and code used in this study are available at [59]. Supplementary material is available online [127].
